# Association between Polymorphisms of Alpha-Adducin Gene and Essential Hypertension in Chinese Population

**DOI:** 10.1155/2013/451094

**Published:** 2012-12-20

**Authors:** Li-na Zhang, Lin-dan Ji, Li-juan Fei, Fang Yuan, Yue-miao Zhang, Jin Xu

**Affiliations:** ^1^Department of Preventive Medicine, School of Medicine, Ningbo University, Ningbo 315211, China; ^2^Department of Biochemistry, School of Medicine, Ningbo University, Ningbo 315211, China; ^3^State Key Laboratory of Genetic Resources and Evolution, Kunming Institute of Zoology, Chinese Academy of Sciences, Kunming 650223, China

## Abstract

The association between polymorphisms of **α**-adducin (*ADD1*) gene and essential hypertension is still unclear. Thus, we carried out a case-control study and an interaction analysis to test whether *ADD1* is a common candidate gene for hypertension in the Chinese population. Blood samples and information including body mass index (BMI), smoking habit, and alcohol abuse were collected. Meanwhile, total cholesterol, high density lipoprotein, triglyceride were measured by automatic biochemistry analyzer. All 6 tag single nucleotide polymorphisms (tagSNPs) within *ADD1* gene were genotyped by SNPstream genotyping system. Multifactor dimensionality reduction (MDR) was used to identify the interactions among the SNPs and the non-genetic factors. Results showed that plasma triglyceride, total cholesterol, and BMI were significantly higher in the hypertensive group than in the control group. Result from genotyping indicated that rs4963 was significantly associated with essential hypertension. After stratification by gender, rs4963 was associated with essential hypertension only in males. MDR analysis indicated that interaction among BMI, rs4963, and rs16843452 were involved in susceptibility of hypertension. The present study indicated that rs4963 within *ADD1* gene was associated with essential hypertension in Chinese population, which might be related to altered exonic splicing and disrupted gene regulation.

## 1. Introduction

Adducin (ADD) is a heterodimeric cytoskeleton protein consisting of an *α*-subunit with either a *β*- or a *γ*-subunit [1], and they are encoded by three different genes *ADD1*, *ADD2*, and *ADD3*, respectively. The *α*-subunit is known to increase renal sodium reabsorption and may be involved in the pathophysiology of essential hypertension [2]. Therefore, it is considered as a major candidate gene for essential hypertension. Not surprisingly, a number of studies have investigated the association of *α*-adducin gene (*ADD1*) polymorphisms with essential hypertension in the past two decades.

Human *ADD1* gene is located on chromosome 4p16.3 and comprises 16 exons. One well-studied polymorphism in *ADD1 *gene is a substitution of Gly for Trp at amino acid residue 460 (G460W, rs4961), which was first described by Cusi et al. [3]. *ADD1 *W460W was found to be associated with a form of low renin salt-sensitive hypertension in Caucasians, with reduced response to pressure natriuretic function and with increased proximal sodium reabsorption [4, 5]. Therefore, although a great number of similar studies have been conducted, the results are often inconsistent.

To convince the association of this polymorphism with essential hypertension, several meta-analyses were recently published [6–10]. Most of these analyses failed to provide evidence for the genetic association of *ADD1 *G460W polymorphism with essential hypertension in global population; but it is suggested that the W carrier might increase the risk of essential hypertension in Han Chinese population [7]. However, most studies focus on *ADD1 *G460W polymorphism, and whether other single nucleotide polymorphisms (SNPs) in *ADD1 *gene were associated with essential hypertension is not clear. Therefore, we carried out a case-control study and an interaction analysis to verify whether *ADD1* gene is associated with hypertension in the Chinese population.

## 2. Materials and Methods

### 2.1. Population for Case-Control Study

The participants were chosen from our established community-based epidemiology study of common diseases. We have collected more than 10,000 health records. Subsequently, participants who fulfilled the following criteria were put into our database: 30 to 75 years old, Han Chinese, living in Ningbo City (East Coast of China) for at least three generations and do not have a migration history. The hypertensive subjects and control subjects were matched for age and sex. In addition, only subjects who do not have other cardiovascular disease or major chronic illnesses according to their health records were included in the control group. Lastly, 905 essential hypertension cases and 905 healthy controls, including 392 males and 513 females in each group, were involved in the following studies.

### 2.2. Blood Pressure and Clinical Parameters

Blood pressure measurements were conducted in the morning after the participant had been in the sitting position for 10 minutes. Three BP measurements were obtained at 5-minute intervals using standard mercury sphygmomanometer, and the average of last two measurements was taken as the BP for that participant. Hypertension in this study is defined as a sitting systolic blood pressure (SBP) ≥ 140 mmHg and/or a diastolic blood pressure (DBP) ≥ 90 mmHg, or self-reported use of antihypertension medication (also confirmed by health records). Patients with secondary hypertension were excluded. Normal blood pressure is defined with SBP ≤ 120 mmHg and DBP ≤ 80 mmHg.

Blood samples were collected with informed consent. Subsequently, total cholesterol (TC), high density lipoprotein (HDL), and triglyceride (TG) were measured from these blood samples by Hitachi automatic biochemistry analyzer 7100. Clinical information including body mass index (BMI), and weekly alcohol and cigarettes consumption were also obtained. In this study, who drank ≥70 g alcohol per week for more than 1 year were defined as individuals with alcohol abuse. Moreover, who smoked ≥70 cigarettes per week for more than 1 year were defined as individuals with smoking habit. The protocol of this study was reviewed and approved by the Ethics Committees of Ningbo University.

### 2.3. SNP Genotyping

All six tagSNPs were retrieved from HapMap (http://hapmap.ncbi.nlm.nih.gov/), with tagger pairwise method in CHB: *R* Square cut off = 0.8 and MAF cut off = 0.1. Genomic DNA was obtained from the whole blood using standard phenol/chloroform extraction. Genotyping was performed using the GenomeLab SNPstream Genotyping System (Beckman Coulter Inc.) according to the manufacturer's protocol [11].

### 2.4. Statistical Analysis

Continuous variables are presented as the mean ± SD and analyzed by *t*-test between two groups. Statistical analyses of allele and genotype frequencies between hypertensive cases and healthy controls and between different sex groups were performed by chi-squared test (SPSS 16.0, SPSS Inc.). Hardy-Weinberg equilibrium (HWE) was tested by the software PEDSTATS V0.6.8 (http://www.sph.umich.edu/csg/abecasis/). Linkage equilibrium (LD) blocks were defined based on the confidence intervals method [12] and the most likely haplotypes within each block for individuals were inferred using the Haploview software [13]. Multifactor dimensionality reduction (MDR) was used to identify and characterize interactions among the SNPs and the nongenetic factors, including BMI, serum HDL, TC, and TG level, as well as percentage of smoking and alcohol abuse [14]. The software used for MDR is distributed in a JAVA platform with a graphical user interface and is freely available (http://www.epistasis.org/mdr.html).

All tests were two sided, and *P* values less than 0.05 were considered statistically significant.

## 3. Results

The baseline characteristics of our study population are summarized in Table 1. Age and sex distribution, HDL, smoking percentage, and percentage of alcohol abuse showed no difference between hypertensive and control groups. However, TG, TC, and BMI were significantly higher in the hypertensive group than in the control group (*P* < 0.05).

**Figure 1:**
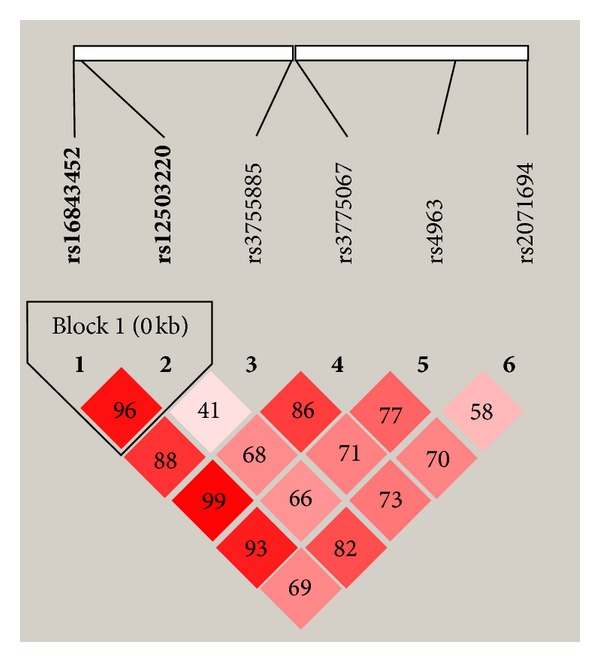
The LD block of 6 tagSNPs within *ADD1* gene. Pairwise linkage disequilibrium (LD) coefficients *D*′ × 100 are shown in each cell. Only one LD block (including rs16843452 and rs12503220) is identified by confidence intervals method.

Genotype distributions of six tagSNPs within *ADD1 *gene were shown in Table 2. None of them deviated from the HWE (*P* > 0.05). Only rs4963 was significantly associated with essential hypertension (*P* = 0.02, odds ratio (OR) = 0.85, 95% confident interval (CI) = 0.75–0.97). After stratification by gender, rs4963 was associated with essential hypertension only in males, and the other 5 SNPs were still negative (Table 3). We further performed haplotype analysis within LD blocks of *ADD1*. Only one LD block and three common haplotypes within the LD block (including rs16843452 and rs12503220) were identified (Figure 1). Of these three haplotypes, no haplotype was significantly associated with hypertension (Table 4).

 Finally, MDR was used to analyze the interaction among different SNPs and nongenetic risk factors for hypertension. After input the genotypes of 6 SNPs together with information about TG, TC, HDL, BMI, smoking, and alcohol abuse, the software outputs the best model for “BMI, rs4963, rs16843452” with 10/10 crossvalidation consistency (Table 5).

## 4. Discussion

Among the three adducin genes, *ADD1 *has received more attention than the other two due to its association with several diseases, such as hyperlipidemia [15], renal disease [16], and coronary heart disease [17]. Series of studies in humans reveal that mutation of *ADD1 *gene may lead to the stimulation of the sodium and potassium-adenosine triphosphatase (ATPase) activity in renal tubular cells, increased renal sodium reabsorption, and subsequently hypertension [18, 19]. 

Since hypertension is considered polygenic, resulting from the interaction of several genes and together with environmental factors, a single SNP has weak effects on the phenotype. A family study from van Rijin et al. [20] showed that *ADD1 *G460W polymorphism was able to explain only a very minute proportion of the heritability of BP traits. Therefore, it is necessary to explore whether there are any other SNPs within *ADD1 *gene that are also associated with essential hypertension. In order to answer this question, we carried out a case-control study in the Chinese population. The result showed that rs4963 was associated with essential hypertension, especially in males. One possible reason for this result might be that men have higher TG (1.93 versus 1.69 mM) and TC (5.39 versus 5.08 mM) level than women.

Out of 1,113 SNPs associated with *ADD1* gene [21], 9 are identified to be nonsynonymous by functional single-nucleotide polymorphism (F-SNP) database [22]. rs4963 is one of them, and the amino acid change found is from Serine to Cysteine. The G allele of rs4963 has deleterious effect by disrupting the activity of exonic splicing and thus disrupt proper gene regulation [23]. In current study, G allele of rs4963 increases the susceptibility of hypertension, which might be associated with altered protein structure and function.

Moreover, environmental factors and individual's biological characteristics, including excess dietary sodium intake, alcohol abuse, smoking, obesity, stress, BMI, TC, and TG, cannot be neglected [24, 25]. We also found interesting interactions between genetic factors and nongenetic risk factors. High BMI and serum TG level were already confirmed to be risk factors for hypertension [26]. The MDR analysis in this study demonstrated that these important risk factors interacted with the genetic factor. Thus, the present interaction analysis gave a little more information than the single genetic study.

In conclusion, the present study indicated that rs4963 within *ADD1 *gene was associated with essential hypertension in Chinese population, which might be related to altered exonic splicing and disrupted gene regulation. Regulation of sodium transport is such a complex process that at least 189 genes are involved according to gene ontology (GO: 0002028). *ADD1 *is only a common member of this system, and polymorphisms within this gene may play a tiny role. Other genes involved in sodium transport regulation system need to be studied. In addition, in our interaction analysis, a significant interaction was found between genetic and nongenetic factors, demonstrating that genetic study alone is not a sufficient indicator for hypertension. To decipher causal factors leading to the development and the pathogenesis of hypertension, future work will require analysis of gene-environmental interaction.

## Figures and Tables

**Table 1:** Baseline characters of the investigated participants.

Variables	Hypertensive	Control	*P* value
Number	905	905	N/A
Sex (male) (%)	43.3	43.3	N/A
Age (y)	56.91 ± 7.37	56.60 ± 7.51	*P* = 0.38
TG (mM)	2.02 ± 1.68	1.63 ± 1.12	*P* < 0.01
HDL (mM)	1.41 ± 0.35	1.41 ± 0.32	*P* = 0.72
TC (mM)	5.34 ± 1.00	5.17 ± 0.93	*P* < 0.01
BMI (Kg/m^2^)	24.65 ± 3.24	23.21 ± 2.86	*P* < 0.01
Regular smoking	173	147	*P* = 0.11
Regular alcohol drinking	152	148	*P* = 0.80

TG: triglyceride; HDL: high density lipoprotein; TC: total cholesterol; BMI: body mass index.

**Table 2:** Association analysis of 6 tagSNPs.

SNP	Group	Genotype			*P* value	OR	95% CI
rs16843452	Hypertensive	243 (CC)	462 (CT)	195 (TT)	0.89	1.01	0.89~1.15
	Control	243 (CC)	461 (CT)	199 (TT)			
rs12503220	Hypertensive	11 (AA)	253 (AG)	636 (GG)	0.26	0.90	0.75~1.08
	Control	22 (AA)	257 (AG)	624 (GG)			
rs4963	Hypertensive	192 (CC)	432 (CG)	278 (GG)	0.02	0.85	0.75~0.97
	Control	224 (CC)	441 (CG)	239 (GG)			
rs3755885	Hypertensive	62 (CC)	379 (CG)	461 (GG)	0.24	0.92	0.79~1.06
	Control	67 (CC)	402 (CG)	435 (GG)			
rs2071694	Hypertensive	447 (CC)	374 (CG)	82 (GG)	0.81	0.98	0.85~1.13
	Control	455 (CC)	366 (CG)	83 (GG)			
rs3775067	Hypertensive	318 (AA)	435 (AG)	148 (GG)	0.27	1.08	0.94~1.23
	Control	287 (AA)	469 (AG)	149 (GG)			

OR: odds ratio; CI: confidence interval.

**Table 3:** Association analysis of 6 tagSNPs in men and women.

SNP	Group	*P* value	OR	95% CI
rs16843452	Male	0.51	0.94	0.77~1.14
	Female	0.45	1.07	0.90~1.27
rs12503220	Male	0.31	0.87	0.66~1.14
	Female	0.53	0.93	0.73~1.17
rs4963	Male	0.02	0.79	0.65~0.97
	Female	0.25	0.90	0.76~1.07
rs3755885	Male	0.80	1.03	0.83~1.28
	Female	0.08	0.84	0.69~1.02
rs2071694	Male	0.28	0.89	0.72~1.10
	Female	0.53	1.06	0.88~1.29
rs3775067	Male	0.21	1.14	0.93~1.40
	Female	0.71	1.03	0.87~1.23

OR: odds ratio; CI: confidence interval.

**Table 4:** Association analysis of haplotypes with hypertension.

Haplotype sequence	Haplotype frequency	Case/control ratio	*P* value
TG	0.472	0.473, 0.470	0.840
CG	0.368	0.360, 0.377	0.274
CA	0.157	0.165, 0.149	0.208

**Table 5:** MDR analysis of gene-environment interaction.

Best model	Testing accuracy	Testing sensitivity	Testing odds ratio	Testing *χ* ^2^	Crossvalidation consistency
BMI	0.59	0.43	2.26 (95% CI: 1.20–4.27)	6.50 (*P* = 0.010)	10/10
BMI, rs4963, rs16843452	0.61	0.54	2.42 (95% CI: 1.33–4.44)	8.40 (*P* = 0.004)	10/10
BMI, TG	0.58	0.57	1.98 (95% CI: 1.10–3.58)	5.18 (*P* = 0.023)	7/10
